# Clinical Relevance of Novel Polymorphisms in the Dihydropyrimidine Dehydrogenase (*DPYD*) Gene in Patients with Severe Fluoropyrimidine Toxicity: A Spanish Case-Control Study

**DOI:** 10.3390/pharmaceutics13122036

**Published:** 2021-11-29

**Authors:** Paula Soria-Chacartegui, Gonzalo Villapalos-García, Luis A. López-Fernández, Marcos Navares-Gómez, Gina Mejía-Abril, Francisco Abad-Santos, Pablo Zubiaur

**Affiliations:** 1 Clinical Pharmacology Department, La Princesa University Hospital, Instituto Teófilo Hernando, Instituto de Investigación Sanitaria La Princesa (IP), Universidad Autónoma de Madrid (UAM), 28029 Madrid, Spain; paulasch98@gmail.com (P.S.-C.); g.villapalos@salud.madrid.org (G.V.-G.); marcos.navares@salud.madrid.org (M.N.-G.); ginapaola.mejia@scren.es (G.M.-A.); 2 Hospital General Universitario Gregorio Marañón, Instituto de Investigación Sanitaria Gregorio Marañón (IiSGM), 28007 Madrid, Spain; llopezf.hgugm@gmail.com; 3 Centro de Investigación Biomédica en Red de Enfermedades Hepáticas y Digestivas (CIBERehd), Instituto de Salud Carlos III, 28029 Madrid, Spain

**Keywords:** dihydropyrimidine dehydrogenase (*DPYD*), capecitabine, 5-fluorouracil, polymorphism, pharmacogenetics

## Abstract

Among cancer patients treated with fluoropyrimidines, 10–40% develop severe toxicity. Polymorphism of the dihydropyrimidine dehydrogenase (*DPYD*) gene may reduce DPD function, the main enzyme responsible for the metabolism of fluoropyrimidines. This leads to drug accumulation and to an increased risk of toxicity. Routine genotyping of this gene, which usually includes *DPYD* *HapB3, *2A, *13 and c.2846A > T (D949V) variants, helps predict approximately 20–30% of toxicity cases. For DPD intermediate (IM) or poor (PM) metabolizers, a dose adjustment or drug switch is warranted to avoid toxicity, respectively. Societies such as the Spanish Society of Pharmacogenetics and Pharmacogenomics (SEFF), the Dutch Pharmacogenetics Working Group (DPWG) or the Clinical Pharmacogenetics Implementation Consortium (CPIC) and regulatory agencies (e.g., the Spanish Medicines Agency, AEMPS) already recommend *DPYD* routine genotyping. However, the predictive capacity of genotyping is currently still limited. This can be explained by the presence of unknown polymorphisms affecting the function of the enzyme. In this case-control work, 11 cases of severe fluoropyrimidine toxicity in patients who did not carry any of the four variants mentioned above were matched with 22 controls, who did not develop toxicity and did not carry any variant. The *DPYD* exome was sequenced (Sanger) in search of potentially pathogenic mutations. *DPYD* rs367619008 (c.187 A > G, p.Lys63Glu), rs200643089 (c.2324 T > G, p.Leu775Trp) and rs76387818 (c.1084G > A, p.Val362Ile) increased the percentage of explained toxicities to 38–48%. Moreover, there was an intronic variant considered potentially pathogenic: rs944174134 (c.322-63G > A). Further studies are needed to confirm its clinical relevance. The remaining variants were considered non-pathogenic.

## 1. Introduction

Fluoropyrimidines constitute a family of drugs widely used in oncology for the inhibition of tumor growth [1], which include capecitabine, tegafur and 5-fluorouracil (5-FU). They are indicated for the treatment of a variety of solid tumors, such as breast, colorectal, and aerodigestive tract cancers [2] and head and neck tumors [3,4,5]. Capecitabine and tegafur are prodrugs of 5-FU, and therefore, they need to be metabolized by different enzymes to form 5-FU. The latter is transformed by various enzymes into active metabolites, such as fluorodeoxyuridine monophosphate (FdUMP), fluorodeoxyuridine triphosphate (FdUTP) and fluorouridine triphosphate (FUTP) [6]. The main mechanism of action of this family of drugs is the inhibition of thymidylate synthase (TYMS). This enzyme methylates deoxyuridine monophosphate (dUMP), producing deoxythymidine monophosphate (dTMP). The metabolite, FdUMP, covalently binds to TYMS enzyme at the dUMP binding site, preventing dUMP from binding and thereby preventing it from being methylated and producing dTMP. Thus, deoxythymidine triphosphate (dTTP) levels decrease and deoxyuridine triphosphate (dUTP) levels increase, which, by interacting with different metabolic pathways, lead to an imbalance of the other nucleotides. In addition, when there is an excess of dUTP, it is incorrectly incorporated into the DNA, generating mismatches which ultimately lead to cell damage and death. Other mechanisms of action were described, consisting of the direct incorporation of metabolites, such as FdUTP and FUTP, into DNA and RNA, respectively. This incorporation causes increased DNA repair by base excision, leading to DNA fragmentation and cell death [6]. The dihydropyrimidine dehydrogenase (DPD), encoded by the *DPYD* gene, metabolizes 5-FU to dihydro-5-fluorouracil (DHFU). Dihydropyrimidinase (DHP) acts on this compound, cleaving the pyrimidine ring and producing 5-fluoro-ureidopropionic acid (FUPA). FUPA is metabolized to α-fluoro-β-alanine (FBAL) by the β-ureido-propionase enzyme (BUP1), and FBAL is excreted in the urine. The DPD enzyme is the limiting factor in this pathway that regulates the cytosolic accumulation of 5-FU [5,6], as 85% of the fluoropyrimidine dose administered is metabolized via this enzyme [7].

The main disadvantage of fluoropyrimidines is their narrow therapeutic range; approximately 10–40% of the patients treated with 5-FU develop severe toxicity [1]. Among the most frequent adverse drug reactions (ADRs) are skin toxicity, including hand-foot syndrome, digestive toxicity, including emesis, diarrhea, enterocolitis and mucositis, cardiac toxicity and hematological toxicity, neutropenia being the most worrisome [1,2,8]. These toxicities are often associated with a partial or total reduction of DPD activity, leading to the accumulation of the drug within the organism by reducing its metabolism and, thus, its excretion [2].

With regards to DPD activity reduction, numerous single nucleotide polymorphisms (SNPs) are described in *DPYD*, many of which cause such reduction in the enzyme’s function. The most frequent ones with impact on the enzyme’s function are: *2A, *13, *HapB3 and rs67376798 [9]. In December 2013, the Clinical Pharmacogenetics Implementation Consortium (CPIC) published the first version of their guideline on fluoropyrimidine dosing and *DPYD* genotype [10], which was updated in October 2017 [11]. The complete changelog for this publication can be access here: https://cpicpgx.org/guidelines/guideline-for-fluoropyrimidines-and-dpyd/, accessed on 9 November 2021. Briefly, after the 2018s update, intermediate metabolizers with an activity score (AS) of 1.5 (i.e., heterozygous carriers of decreased-function alleles, such as *1/*HapB3 [rs75017182]), as well as those with AS = 1.0 (i.e., heterozygous carriers of no-function alleles, such as *2A [rs3918290] or homozygous carriers of decreased-function alleles, such as c.2846A > T [rs67376798]) require a dose reduction of 50% “followed by dose titration, based on clinical judgement and ideally therapeutic drug monitoring”. Moreover, poor metabolizers (PMs) with an AS = 0.5 should avoid 5-FU based regimens or, in case alternative agents are not suitable, a 25% of the recommended initial dose may be administered. Lastly, PMs with AS = 0 may not receive fluoropyrimidines. The frequency of IM is 3–5% and that of PM is 0.2% [1]. Furthermore, currently, the Spanish Drugs Agency (AEMPS) recommends the genotyping of the following variants prior fluoropyrimidine prescription: *2A, *13, *HapB3 and rs67376798 [9]. However, there is no complete correlation between the presence of the latter variants and the occurrence of toxicity [12]. Other factors may be responsible for toxicity, such as a patient’s age, general condition, co-morbidities, as well as polymorphism of other genes, among others [13]. In fact, the Spanish Society of Pharmacogenetics and Pharmacogenomics (SEFF) will soon publish their Guideline on *DPYD* genotyping and prescription of fluoropyrimidines, where genotyping of the four core variants is considered mandatory and the genotyping of six additional variants is recommended [14]. Ultimately, the functional impact of several additional *DPYD* SNPs remains unknown to date or, further, several variants may have not been described yet. Both of them could be responsible for the reduction in DPD activity and, consequently, of the occurrence of fluoropyrimidine toxicity [15]. Therefore, the aim of this work was to sequence the *DPYD* gene in patients treated with fluoropyrimidines, managed at Hospital Universitario de La Princesa, who presented severe toxicity and who did not carry any of the above-mentioned alleles. This work is part of La Princesa Multidisciplinary Initiative for the implementation of pharmacogenetics (PriME-PGx) [16].

## 2. Materials and Methods

### 2.1. Study Procedures and Population

The present work was designed as an observational, retrospective case-control study. The study protocol was approved by the Hospital’s Research Ethics Committee (registration number: 4358, 28 December 2020). Participants were patients with breast or digestive tract cancer who were treated with capecitabine and/or 5-FU, in monotherapy or as part of a regimen since 2013 to 2020. All patients gave informed consent for the *DPYD* genetic study to their oncologist for care reasons and were genotyped for the four *DPYD* variants recommended by the AEMPS: *DPYD* *2A, *13, rs67376798 and *HapB3. Only those individuals who did not carry any of these variants were included in the study. For each patient, the sex, age, type of disease, the drug received and the presence or absence of toxicity, as well as its degree of severity, were recorded.

The classification into cases and controls was based on the degree of toxicity suffered according to the Common Terminology Criteria for Adverse Events (CTCAE) scale [17]. For this purpose, patient’s medical records were reviewed and the episodes of toxicity suffered during the first two treatment cycles were recorded. Those individuals who showed an episode of toxicity grade III or higher in any of the first two treatment cycles were included as cases, and those who did not were classified as controls. Cases were matched with two controls each, according to five factors: the drug received, chemotherapy regimen, sex, age and type of oncological disease. Finally, 11 cases and 22 controls were included.

### 2.2. Sequencing

Stored DNA aliquots at −80 °C were used for sequencing. When not available, blood tubes were recovered and DNA was extracted in a Maxwell RSC automated extractor (Promega Biotech Ibérica, Alcobendas, Spain). The amplification of the 23 *DPYD* exons was accomplished in a SimpliAmp thermal cycler (Applied Biosystems, ThermoFisher Scientific, Waltham, MA, USA). For this purpose, 23 pairs of primers were used, specific for the intronic regions adjacent to the beginning and end of each exon (Supplementary Table S1). Following the manufacturer’s instructions, the ExoSAP-It reagent (Applied Biosystems, ThermoFisher Scientific, USA) was used to purify the amplified PCR product. Afterwards, Sanger sequencing was outsourced at the Genomics Unit of the Gregorio Marañón General University Hospital. The SnapGene version 5.3 software was used for sequence analysis. The sequences obtained were aligned with the reference sequence of the *DPYD* gene from GenBank (GRCh38.p13) and mismatches were noted.

### 2.3. Statistical Analysis

The SPSS software version 23.0 was used (SPSS Inc., Chicago, IL, USA). A Student’s *t*-test was performed to test for significant differences in age between cases and controls. In addition, a Chi-squared test was performed to check for differences in sex, drug received, strategy followed and disease between cases and controls. Finally, a Chi-squared or Fisher exact test were used to analyze whether there were significant differences in the presence of each of the identified variants between cases and controls. A type 1 error of α = 0.05 was assumed; the significance level established in all analyses was *p* < 0.05.

## 3. Results

### Study Population

Baseline characteristics of the study population are shown in Table 1. A good match between cases and controls was confirmed as no significant differences between them in terms of age, sex, drug received, strategy followed and disease were observed. Females accounted for an 81.8% of both cases and controls. Capecitabine and combined regimes were more frequent than 5-FU and monotherapy, respectively. The large bowel was the organ more frequently affected (Table 1). The individual description of demographics, disease, treatments and types of toxicity reported in the 11 cases of the present study is shown in Table 2.

Table 3 shows the variants observed in the 11 cases and 22 controls; Table 4 shows genotype and allele frequencies in cases and controls. A total of 17 different SNPs were observed. Among them, six are currently acknowledged in CPIC’s *DPYD* allele definition tables, all of them considered normal function variants. Three exonic variants were exclusively found in two cases: rs367619008 (c.187A > G, p.Lys63Glu), rs200643089 (c.2324T > G, p.Leu775Trp) and rs76387818 (c.1084G > A, p.Val362Ile). The last two (rs200643089 and rs76387818) were observed in the same case. An intronic variant was exclusively observed in one case: c.322-63G > A (rs944174134). Furthermore, another intronic variant, 1740 + 39C > T (rs2786783), showed a prevalence of 13.63% in cases compared to 2.27% in controls (*p* = 0.035). This variant was linked to the *5 allele (D’ = 1, R^2^ = 0.9359). No other variant was significantly more prevalent in cases compared to controls.

## 4. Discussion

Two million cancer patients are estimated to be treated with fluoropyrimidines annually [18] and 10–40% of these develop severe toxicity [1]. Severe toxicity caused by antitumor drugs increases healthcare costs [19] and may generate adherence problems, and even drug discontinuation, which can affect the effectiveness of pharmacotherapy [20]. Naturally, this is of great concern, given the severity of a disease such as cancer not being adequately treated. For this reason, it is necessary to determine the genetic, demographic or clinical factors that predispose to fluoropyrimidine toxicity in order to, when possible, avoid or reduce it. Clinical pharmacogenetics is one strategy to reduce these toxicities by avoiding prescribing fluoropyrimidines to patients who cannot metabolize them correctly. In this line, the evidence that pre-emptive genotyping leads to a reduction in the incidence of adverse reactions is indisputable, as several institutions, such as the CPIC, SEFF or the Dutch Pharmacogenetics Working Group (DPWG), already recommend it [10,14,21]. Furthermore, since 2020, the AEMPS recommends pre-emptive genotyping of *DPYD* *HapB3, *2A, *13 and c.2846A > T (D949V) [9]. These four SNPs could be considered the basic set of polymorphisms to be genotyped in routine clinical practice. However, the sensitivity of *DPYD* genotyping, when combining the latter four variants is only 20–30% [11]. Consistently, since 2013 to 2020, severe fluoropyrimidine-induced toxicity occurred in 11 patients which had tested negative for the four variants. In conclusion, all this suggests that additional variants in the *DPYD* gene may impair DPD activity and, consequently, fluoropyrimidines accumulate and, subsequently, toxicity is evidenced.

Among the 11 studied cases, two showed *DPYD* genotypes consistent with the development of toxicities. The first case carried the rs367619008 (c.187A > G, p.Lys63Glu) variant (in heterozygosis). In Europeans, this variant shows a frequency of <0.01% [22]. The lysine at this position contributes to the stabilization of the protein’s FAD-binding site through interactions with other amino acids. When a glutamate occupies the lysine position, the interactions that stabilize the domain are modified, altering electron transport and, thus, protein function [23]. Three studies in the literature previously associated this variant with a decrease in DPD activity, which did not appear in control individuals or in individuals with low toxicity [15,23,24], which is consistent with our findings. Therefore, we suggest that this variant is clinically relevant and should be genotyped prior to fluoropyrimidine prescription.

The second case showed the rs200643089 (c.2324T > G, p.Leu775Trp) and rs76387818 (c.1084G > A, p.Val362Ile) variants. The first variant shows a frequency of 0.003% in the global population; the second has a frequency of 3.2% in Europeans. To our knowledge, only one study previously analyzed the first variant, present in a patient with toxicity and, therefore, proposed to cause an alteration of DPD function by being in close proximity to the 5-FU binding site [25]. In the latter work, also performed in a Spanish population, both variants (rs200643089 and rs76387818) were similarly observed in the one case. This suggests that these SNPs could be in disequilibrium linkage and the allele containing both SNPs may be responsible for the toxicity, which has a non-negligible frequency in our population. In addition, the carrier patient was phenotyped by measuring plasma Uracil concentration and was confirmed as DPD partially deficient. Concerning rs76387818, this variant was related solely in a previous work to toxicity [26], while, in other work, this association was not observed [27]. Should this variant be a decreased-function variant like *HapB3, it would be expected that some heterozygous subjects did not suffer toxicity, as the DPD activity (activity score of 1.5) could be sufficient in some patients to metabolize the drug to a considerable extent. In contrast, the allele formed by rs200643089 and rs76387818 could be a no-function allele, which would explain the toxicity observed in our study population and that of the other study with a Spanish population [25]. Alternatively, both variants could be located in different alleles, patients would be IMs with an AS of 1 or less, being higher the risk for toxicity. Regardless if these variants occur in the same allele or not, they can be considered potentially pathogenic. Hence, we suggest that genotyping them preemptively may reduce the incidence of ADRs in patients prescribed with fluoropyrimidines.

Two other intronic variants were associated with the development of toxicity; however, due to the nature of the variants (intronic, non-exonic) and the literature support, our conclusions are less compelling. The first of them is c.322-63G > A (rs944174134), which was present in heterozygosis in one case exclusively. To the best of our knowledge, this is the first work that finds this variant in the context of fluoropyrimidine treatment and relates it to the development of toxicity. The second one was c.1740 + 39C > T (rs2786783), which was more prevalent in cases than in controls. Previous works evaluated this variant, showing controversial findings: in two of them, this variant, along with other variants, was related to the development of toxicity [28,29], while in the other, this association was not observed [30]. Both variants were evaluated with the SpliceAI [31] and RegSNPs-intron [32] tools; however, they were considered benign (i.e., neither of them were considered to be acceptor/donor loss/gain variants). Here, this variant was linked to the *5 allele, which would constitute a suballele; therefore, this variant may not be really pathogenic and the differential prevalence of the *5 sub-allele may be spurious.

Overall, as mentioned earlier, the prospective genotyping of *DPYD* core variants only prevents 20–30% of toxicities, since, among the remaining 70–80%, this tool has no predictive power for two reasons: first and foremost, because most of these toxicities are not related to DPD activity/*DPYD* polymorphism, and second, because some of them may be related to unknown polymorphisms. Therefore, among the eleven cases, nine (82%) belong to the first group and two to the second (18%). Considering that at least two of them exhibited pathogenic or potentially pathogenic variants (i.e., 18%), the percentage of toxicities explained would have been 38–48% if rs367619008, rs200643089 and rs76387818 had been genotyped prospectively.

The remaining variants were considered non-pathogenic, based on the literature evidence and the results obtained, since their presence in the controls helps to rule out that they cause lower DPD enzyme activity. These include all the variants identified which were defined as normal function variants by CPIC. The variants rs2297595, rs3790387, rs2811202 and rs56293913 appeared together in four controls, all of them in heterozygosity. They are in linkage disequilibrium, appearing together in 11.21% of the Iberian population, thus probably defining a previously unidentified allele [33]. None of them, nor the hypothetical allele, are likely to be pathogenic.

### Limitations

The main limitation of this study is its retrospective nature and the reduced sample size. Hence, our findings and the interpretation of them should be considered with caution; confirmatory studies are required, preferably prospective and with large sample sizes

## 5. Conclusions

Only 20–30% of the cases of toxicity in patients prescribed fluoropyrimidines can be explained by the four basic variants of *DPYD*: *HapB3, *2A, *13 and c.2846A > T (D949V). In this work, rs367619008 (c.187A > G, p.Lys63Glu), rs200643089 (c.2324T > G, p.Leu775Trp) and rs76387818 (c.1084G > A, p.Val362Ile) variants increased the percentage of the explained toxicities to 38–48%. Further studies are warranted to confirm the clinical relevance of the intronic variants. The remaining variants were considered non-pathogenic, including the identified allele formed by the combination of rs2297595, rs3790387, rs2811202 and rs56293913 variants.

## Figures and Tables

**Table 1:** Baseline characteristics of cases and controls.

Title	Cases	Controls	Total	*p*-Value
	(*N* = 11)	(*N* = 22)	(*N* = 33)	
Age	63.64 (11.79)	64.77 (11.92)	64.39 (11.70)	0.764
Sex				1.000
Males	2 (18.2%)	4 (18.2%)	6 (18.2%)	
Females	9 (81.8%)	18 (81.8%)	27 (81.8%)	
Drug				1.000
Capecitabine	7 (63.6%)	14 (63.6%)	21 (63.6%)	
5-FU	4 (36.4%)	8 (36.4%)	12 (36.4%)	
Strategy				0.618
Monotherapy	4 (36.4%)	10 (45.5%)	14 (42.4%)	
Combined	7 (63.6%)	12 (54.5%)	19 (57.6%)	
Carcinoma location				0.313
Breast	3 (27.3%)	6 (27.3%)	9 (27.3%)	
Large bowel	6 (54.5%)	16 (72.7%)	22 (66.7%)	
Stomach	2 (18.2%)	0 (0%)	2 (6%)	

Data are presented as mean (standard deviation) or as count (percentage of total).

**Table 2:** Demographics, disease, treatments and types and severity of toxicity reported in the 11 cases of the present study.

Case	Demographics	Disease	Treatment	Enterocolitis	Neutropenia	Mucositis or Stomatitis	Diarrhoea	Emesis	Cutaneous toxicity	Anorexia	Asthenia
1	65-year-old male	Colon adenocarcinoma	FOLFOX (5-FU)	IV							
2	46-year-old woman	Breast carcinoma	XELOX (Capecitabine)	III	IV						
3	55-year-old woman	Gastric adenocarcinoma	5-FU, epirubicin, cisplatin			IV	IV	IV			
4	78-year-old woman	Colon adenocarcinoma	Capecitabine	III		III		III			
5	47-year-old woman	Rectal adenocarcinoma	XELOX (Capecitabine)	III							
6	79-year-old woman	Gastric adenocarcinoma	XELOX (Capecitabine)			III	III	III			
7	67-year-old woman	Colon adenocarcinoma	Capecitabine	III					III		
8	69-year-old woman	Anal adenocarcinoma	5-FU and cisplatin			III					
9	71-year-old woman	Breast carcinoma	Capecitabine							III	III
10	52-year-old male	Colon adenocarcinoma	FOLFOX (5-FU)		III						
11	71-year-old woman	Breast carcinoma	Capecitabine				IV	IV			

Toxicity severity is shown according to the Common Terminology Criteria for Adverse Events (CTCAE) scale.

**Table 3:** Variants identified in all cases and controls in the present study.

Location	E2	E3	I (E3–E4)	I (E4–E5)	E6		I (E7–E8)	I (E9–E10)	E10	I (E10–E11)	E13	I (E13–E14)	I (E13–E14)	E14	I (E16–E17)	E18	I (E18–E19)	E19
Variant (coding)	c.85T > C	c.187A > G	c.234-81G > A	c.322-63G > C	c.496A > G		c.763-118A > G	c.958 + 134T > G	c.1084G > A	c.1129-15T > C	c.1627A > G	c.1740 + 39C > T	c.1740 + 40A > G	c.1896T > C	c.2058 + 101T > C	c.2194G > A	c.2300-39G > A	c.2324T > G
Variant (protein)	p.Cys29Arg	p.Lys63Glu	N/A	N/A	p.Met166Val		N/A	N/A	p.Val362Ile	N/A	p.Ile543Val	N/A	N/A	p.Phe632Phe	N/A	p.Val732Ile	N/A	p.Leu775Trp
Variant (RefSeq)	rs1801265	rs367619008		rs944174134	rs2297595		rs3790387	rs2811202	rs76387818	rs56293913	rs1801159	rs2786783	rs2811178	rs17376848	rs1890138	rs1801160	rs12137711	rs200643089
CPIC status	NF (Strong)	Not included	Not included	Not included	NF (moderate)		Not included	Not included	Not included	Not included	NF (strong)	NF (strong)	Not included	NF (moderate)	Not included	NF (moderate)	Not included	Not included
Allele	*9A										*5					*6		
Case 1													G/G					
Case 2		A/G														*1/*6		
Case 3													A/G					
Case 4													A/G				G/A	
Case 5	*1/*9A												A/G					
Case 6													A/G					
Case 7											*5/*5	T/T	G/G					
Case 8	*1/*9A										*1/*5		A/G				G/A	
Case 9	*1/*9A			G/C									A/G					
Case 10	*1/*9A												A/G					
Case 11									G/A		*1/*5	C/T	G/G					T/G
Cnt 1	*1/*9A												G/G					
Cnt 2													A/G			*1/*6		
Cn t3	*1/*9A				A/G		A/G	T/G		T/C	*1/*5		G/G				G/A	
Cnt 4													A/G		C/C			
Cnt 5	*1/*9A				A/G		A/G	T/G		T/C	*1/*5		G/G					
Cnt 6													A/G					
Cnt 7													G/G					
Cnt 8															C/C			
Cnt 9													A/G					
Cnt 10	*1/*9A												A/G					
Cnt 11	*1/*9A												A/G					
Cnt 12	*1/*9A		G/A		A/G								A/G					
Cnt 13													A/G				G/A	
Cnt 14													G/G					
Cnt 15	*1/*9A														T/C			
Cnt 16	*9/*9				A/G		A/G	T/G		T/C	*1/*5	C/T	G/G			*1/*6		
Cnt 17													A/G	T/C				
Cnt 18	*1/*9A				A/G		A/G	T/G		T/C	*1/*5			T/C				
Cnt 19													A/G					
Cnt 20													G/G					
Cnt 21					A/G										T/C	*1/*6		
Cnt 22													A/G					

Empty boxes indicate a wild-type genotype. Abbreviations: E: exon; I: intron; Cnt: control; NF: normal function. CPIC status: variant acknowledged in allele definition tables of the Clinical Pharmacogenetics Implementation Consortium guideline on fluoropyrimidines and *DPYD* testing.

**Table 4:** Genotype and allele frequencies of the identified *DPYD* variants among cases and controls.

*DPYD* Variant	Genotype or Allele	Cases	Controls	Total	*p*	*DPYD* Variant	Genotype or Allele	Cases	Controls	Total	*p*
*9A c.85T > C Cys29Arg rs1801265	*1/*1	7 (63.6%)	13 (59.1%)	20 (60.6%)	0.769	c.1129-15T > C rs56293913	TT	11 (100%)	18 (81.8%)	29 (87.9%)	0.131
	*1/*9	4 (36.4%)	8 (36.4%)	12(36.4%)			TC	0 (0%)	4 (18.2%)	4 (12.1%)	
	*9/*9	0 (0%)	1 (4.5%)	1 (3%)			CC	0 (0%)	0 (0%)	0 (0%)	
	*1	18 (81.8%)	34 (77.3%)	52 (78.8%)	0.759		T	22 (100%)	40 (90.9%)	62 (93.9%)	0.380
	*9	4 (18.2%)	10 (22.7%)	14 (21.2%)			C	0 (0%)	4 (9.1%)	4 (6.1%)	
c.187A > G p.Lys63Glu rs367619008	AA	10 (90.9%)	22 (100%)	32 (97.0%)	0.151	c.1627A > G p.Ile543Val rs1801159	TT	9 (81.8%)	18 (81.8%)	27 (81.8%)	0.354
	AG	1 (9.1%)	0 (0%)	1 (3%)			TG	1 (9.1%)	4 (18.2%)	5 (15.2%)	
	GG	0 (0%)	0 (0%)	0 (0%)			GG	1 (9.1%)	0 (0%)	1(3%)	
	A	21 (95.5%)	44 (100%)	65 (98.5%)	0.541		T	19 (86.4%)	40 (90.9%)	59 (89.4%)	0.690
	G	1 (4.5%)	0 (0%)	1 (1.5%)			G	3 (13.6%)	4 (9.1%)	7 (10.6%)	
c.234-81G > A	GG	11 (100%)	21 (95.5%)	32 (97.0%)	0.473	c.1740 + 39 C > T rs2786783	CC	9 (81.8%)	21 (95.5%)	30 (90.9%)	0.301
	GA	0 (0%)	1 (4.5%)	1 (3%)			CT	1 (9.1%)	1 (4.5%)	2 (6.1%)	
	AA	0 (0%)	0 (0%)	0 (0%)			TT	1 (9.1%)	0 (0%)	1(3%)	
	G	22 (100%)	43 (97.7%)	65 (98.5%)	0.688		C	19 (86.4%)	43 (97.7%)	62 (93.9%)	0.035
	A	0 (0%)	1 (2.3%)	1 (1.5%)			T	3 (13.6%)	1 (2.3%)	4 (6.1%)	
c.322-63G > C rs944174134	GG	10 (90.9%)	22 (100%)	32 (97.0%)	0.151	c.1740 + 40A > G rs2811178	AA	1 (9.1%)	4 (18.2%)	5 (15.2%)	0.705
	GC	1 (9.1%)	0 (0%)	1 (3%)			AG	7 (63.6%)	11 (50%)	18 (54.5%)	
	CC	0 (0%)	0 (0%)	0 (0%)			GG	3 (27.3%)	7 (31.8%)	10 (30.3%)	
	G	21 (95.5%)	44 (100%)	65 (98.5%)	0.541		A	9 (40.9%)	19 (43.2%)	28 (42.4%)	0.860
	C	1 (4.5%)	0 (0%)	1 (1.5%)			G	13 (59.1%)	25 (56.8%)	38 (57.6%)	
c.496A > G p.Met166Val rs2297595	AA	11 (100%)	16 (72.7%)	27 (81.8%)	0.056	c.1896T > C p.Phe632Phe rs17376848	TT	11 (100%)	20 (90.9%)	31 (93.9%)	0.302
	AG	0 (0%)	6 (27.3%)	6 (18.2%)			TC	0 (0%)	2 (9.1%)	2 (6.1%)	
	GG	0 (0%)	0 (0%)	0 (0%)			CC	0 (0%)	0 (0%)	0 (0%)	
	A	22 (100%)	38 (86.4%)	60 (90.9%)	0.190		T	22 (100%)	42 (95.5%)	64 (97.0%)	0.980
	G	0 (0%)	6 (13.6%)	6 (9.1%)			C	0 (0%)	2 (4.5%)	2 (3%)	
c.763-118A > G N/A rs3790387	AA	11 (100%)	18 (81.8%)	29 (87.9%)	0.131	c.2058 + 101 T > C rs1890138	TT	11 (100%)	18 (81.8%)	29 (87.8%)	0.320
	AG	0 (0%)	4 (18.2%)	4 (12.1%)			TC	0 (0%)	2 (9.1%)	2 (6.1%)	
	GG	0 (0%)	0 (0%)	0 (0%)			CC	0 (0%)	2 (9.1%)	2 (6.1%)	
	A	22 (100%)	40 (90.9%)	62 (93.9%)	0.380		T	22 (100%)	38 (86.4%)	60 (90.9%)	0.190
	G	0 (0%)	4 (9.1%)	4 (6.1%)			C	0 (0%)	6 (13.6%)	6 (9.1%)	
c.958 + 134T > G rs2811202	TT	11 (100%)	18 (81.8%)	29 (87.9%)	0.131	c.2194G > A p.Val732Ile rs1801160	GG	10 (90.9%)	19 (86.4%)	29 (87.9%)	0.706
	TG	0 (0%)	4 (18.2%)	4 (12.1%)			GA	1 (9.1%)	3 (13.6%)	4 (12.1%)	
	GG	0 (0%)	0 (0%)	0 (0%)			AA	0 (0%)	0 (0%)	0 (0%)	
	T	22 (100%)	40 (90.9%)	62 (93.9%)	0.380		G	21 (95.5%)	41 (93.2%)	62 (93.9%)	0.333
	G	0 (0%)	4 (9.1%)	4 (6.1%)			A	1 (4.5%)	3 (6.8%)	4 (6.1%)	
c.1084G > A p.Val362Ile rs76387818	GG	10 (90.9%)	22 (100%)	32 (97.0%)	0.151	c.2300-39 G > A rs12137711	GG	9 (81.8%)	20 (90.9%)	29 (87.9%)	0.451
	GA	1 (9.1%)	0 (0%)	1 (3%)			GA	2 (18.2%)	2 (9.1%)	4 (12.1%)	
	AA	0 (0%)	0 (0%)	0 (0%)			AA	0 (0%)	0 (0%)	0 (0%)	
	G	21 (95.5%)	44 (100%)	65 (98.5%)	0.541		G	20 (81.8%)	42 (95.5%)	62 (93.9%)	0.684
	A	1 (4.5%)	0 (0%)	1 (1.5%)			A	2 (9.1%)	2 (4.5%)	4 (6.1%)	
c.2324T > G p.Leu775Trp rs200643089	TT	10 (90.9%)	22 (100%)	32 (97.0%)	0.151						
	TG	1 (9.1%)	0 (0%)	1 (3%)							
	GG	0 (0%)	0 (0%)	0 (0%)							
	T	21 (95.5%)	44 (100%)	65 (98.5%)	0.541						
	G	1 (4.5%)	0 (0%)	1 (1.5%)							

## Data Availability

Not applicable.

## Supplementary Materials

The following are available online at https://www.mdpi.com/article/10.3390/pharmaceutics13122036/s1, Table S1. Sequences and size of the forward and reverse primers used for Sanger sequencing of the *DPYD* gene.
