# Supplementary material for: Clinical Relevance of Novel Polymorphisms in the Dihydropyrimidine Dehydrogenase (DPYD) Gene in Patients with Severe Fluoropyrimidine Toxicity: A Spanish Case-Control Study
Source: Pharmaceutics. 2021 Nov 29;13(12):2036. doi: 10.3390/pharmaceutics13122036 (PMC8707980; doi:10.3390/pharmaceutics13122036)
Supplement: Supplementary file 1 [file pharmaceutics-13-02036-s001.zip › pharmaceutics-1462836-supplementary.pdf]

pharmaceutics

MDPI

Supplementary Materials: Clinical Relevance of Novel Polymorphisms in the Dihydropyrimidine Dehydrogenase (*DPYD*) Gene in Patients with Severe Fluoropyrimidine Toxicity: A Spanish Case-Control Study

Paula Soria-Chacartegui, Gonzalo Villapalos-García, Luis A López-Fernández, Marcos Navares-Gómez, Gina Mejía-Abril and Francisco Abad-Santos and Pablo Zubiaur

Table S1. Sequences and size of the forward and reverse primers used for Sanger sequencing of the *DPYD* gene.

Exon	Forward primer	Reverse primer	Size (bp)
1	ACTTGGCTCTCTGGCTGGAGCTT	AAACTTTCCCGCGTCTCTCACTC	234
2	TTAGCCAGGTGTGGTAGCGTAC	TGCCTTACAATGTGTGGAGTG	410
3	TGAGACTTCTGTGACAGCTGTA	CCTCAAGGGAAGTCTCTCCAC	442
4	GGAGTGCCAAAGATGAAACACA	TGGATTTGCTAAGACAAGCTG	362
4	TCCTATGTGTCAAATACTCTGCT	TGGGTATCAACAGAGCACCA	444
6	AGGAGGCATGACTCTAGAAAGG	CCATTAAAAGAAATATTCACAGGGCT	719
7	AGAATGTAGATGTCCTCATGCA	TGCATGACATTTGCTGTTAATC	331
8	AGCCCTTAATAGAACATGTTCCT	TGAAGGCAGTCATTCTTCTGG	374
9	TGCTTACAGATGTTTTCCTCT	ACAATGTGCTGCTGAGCTTG	324
10	TGGAAAACTGCAAGATGCAA	AGCCCTTGAGTATTGACAAAG	312
11	TGGTGAAAGAAAAAGCTGCAT	GTTCTTTTCAATACTTGCCACT	548
12	TGTGTTGTTAACTCCAATATTTCGT	TCAAGCATCCTCCCGCTT	621
13	TTCGGATGCTGTGTTGAAGT	AATGTGTAATGATAGGTCTTGTCAAA	443
14	GCTTTTCTTTGTCAAAAGGAGAC	AGCTTCACATTGTGTGGGTT	409
15	TAATTCCAAAGCCCCAAATG	TTTCTCATGGCAGCTCTTTATTT	346
16	TCAACGGTGAAAGCCTATTG	AGCTTCCCTCATTTTCCACT	318
17	TTTGTCTTGCACGTCTCCAG	AGGATCTTGTGTTTCCAGATCA	437
18	TGAGAAAGTAAAGTTGTGGTAATT	GGGATCATAAAGGGCACAAA	423
19	TCCAGTGACGCTGTCATCA	ACAGGACAGGAAATAAACCTCA	434
20	AGACGGCTACTGATCCATCA	TCTGAAATAGAAACCAAGGCTGA	375
21	CCCATTTTTCTCTTCTCTGAGC	ATGCATGCTTGCCAGTGT	423
22	CTTTCAGAAGACAAACATCTAAGC	CAGAAAATGCTTTCTGCCGTA	402
23	ACGCTAAAATGGGGACATTG	ACATAAGACAACTGGCAGTG	517
